# Amount of Escape Estimation Based on Bayesian and MCMC Approaches for RNA Interference

**DOI:** 10.1016/j.omtn.2019.10.010

**Published:** 2019-10-18

**Authors:** Tian Liu, Yongzhen Pei, Changguo Li, Ming Ye

**Affiliations:** 1 School of Computer Science and Technology, Tiangong University, Tianjin 300387, China; 2 School of Mathematical Sciences, Tiangong University, Tianjin 300387, China; 3 Department of Basic Science, Army Military Transportation University, Tianjin 300387, China; 4 Department of Scientific Computing, Florida State University, Tallahassee, FL, USA

**Keywords:** amount of escape, Bayesian inference, RNA interference, MCMC method, Gillespie algorithm

## Abstract

The amount of short interfering RNA (siRNA) escaping from the endosome has a significant impact on the efficiency of RNAi. In general, the initial injected amount of siRNAs during the experiment is known, and also the amount of siRNAs after the experiment can be revealed by the level of mRNA measured. However, it is impossible to measure the amount of siRNAs that escape from the endosome and really take part in the chemical reaction of RNAi by detecting the biological organism and its tissues. Inspired by the bottleneck effect in the virus, we introduce the Bayesian approach to infer the amount of escape based on a single type and multiple types of siRNA, respectively. With the consideration of the large calculation quantity of the accurate posterior distribution and the unavailable analytic expression of the likelihood function, our article proposes to take samples by the improved Markov chain Monte Carlo (MCMC) method. The article takes the silencing gene of the synthesis of chitin and the interfering multiple target oncogene as numerical examples to show that our improved MCMC method has higher operation efficiency compared to the Bayesian approach. Our research models siRNA endosome escape using statistical methods for the first time. It perhaps provides a theoretical basis to decrease the cost of a biotic experiment for the future and the standardized statistical approaches for the amount of escape estimation.

## Introduction

RNAi refers to a highly conserved biological process that recognizes double-stranded RNA (dsRNA) in the cell to induce the specific degradation of homologous mRNA during evolution.^1^ Endogenously expressed long dsRNA is first cleaved into short interfering RNA (siRNA) by the enzyme, such as Dicer, that is the component of a gene-silencing mechanism, and then the short RNA molecules are exploited as guides to target homologous RNA species.^2^^,^^3^ The specific suppression of gene expression possibly actualizes through injecting or feeding with dsRNA. The introduction of siRNA into insect cells and silencing of target genes expression offer a new potential tool for the biological pest control method.^4^ For example, the RNAi pathway could be applied to reduce the breeding of lepidopteran and coleopteran insect pests via restraining the planta expression,^5^ and Mao et al.^6^ provide a strategy to impair larval tolerance of gossypol by interfering a cotton bollworm RNA. As a highly efficient technology, RNAi has also developed rapidly in the field of infectious disease and tumor gene therapy,^7^^,^^8^ and it can cure humans with various diseases that traditional drugs cannot, such as chronic hepatitis B virus.^9^ In addition, individualized treatment schemes can be designed according to different conditions of patients.

**Figure 1:**
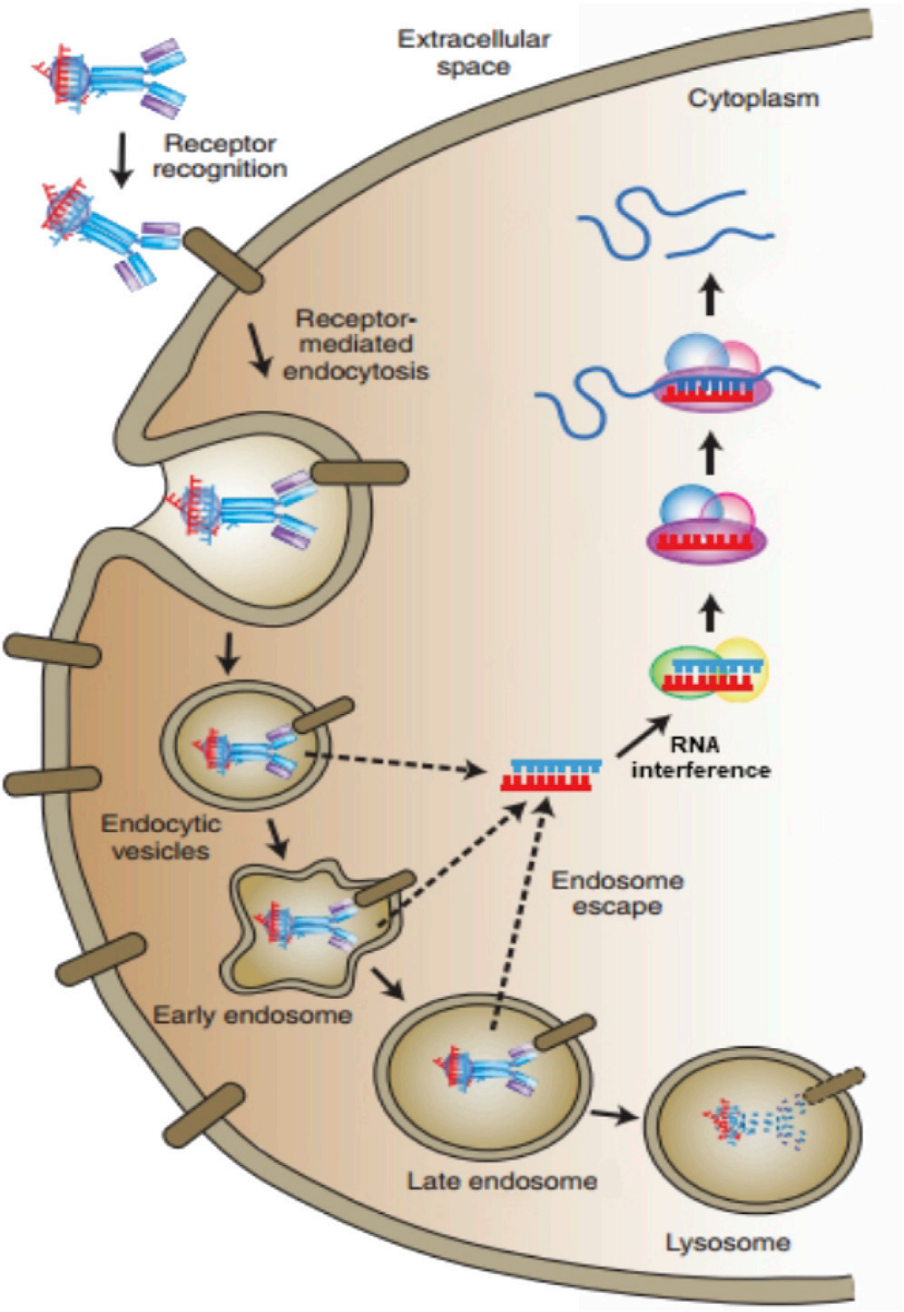
The Process of Escape of siRNA Uptake and intracellular trafficking of a targeted siRNA delivery vehicle.^10^

The significant barrier for efficient siRNA uptake lies in the plasma membrane. In spite of the small size of siRNA molecules, they are still prevented from crossing biological membranes because of their negative charge and hydrophilicity. The procedure of the intracellular transportation of siRNAs begins with early endosomal vesicles. Subsequently, with the fusion of these early endosomes and sorting endosomes, siRNAs are transferred to the late endosomes. Only a small part of siRNAs could escape from the endosomes, and another part with the endosomal contents is removed to the lysosomes. The lysosomes that contain various nucleases acidify the endosomal content, and the siRNAs are degraded in turn. Figure 1 provides a schematic diagram that describes the process of the uptake and intracellular trafficking of a targeted siRNA. So, in order to avert lysosomal degradation, siRNAs have to escape from the endosomes and get into the cytosol, where they will associate with the RNAi mechanism.^10^ Besides, it has been found that some of the generated siRNAs are not directly derived from the cleavage of dsRNA but rather, from a chain reaction of RNA polymerase. With the allowance of a single strand of siRNA as a primer and the target mRNA as a template, this reaction amplifies the target mRNA under the action of RNA-mediated RNA polymerase (RdRP) and generates a new siRNA subpopulation.^11^ These, in turn, would continue to react to the target mRNA and degrade it.^12^ This cyclical amplification process of RNAi explains the reason why a small amount of dsRNA can induce strong gene-silencing effects.

We find that the process of siRNA delivery resembles the biological effect called bottleneck. The bottleneck describes the phenomenon that the number of individuals in a group is reduced drastically or even extinct due to drastic changes in the environment. When we inject a certain amount of siRNA into a pest, only a small fraction of the siRNA can across the plasma membrane and participate in the RNAi, and the remaining siRNAs will be degraded. The lower amount of escaping siRNA (commonly known as bottleneck size) will lead to a form of a new population by the amplification process.^13^ Accurate quantification of the amount of escape for RNAi is vital for several reasons. First, the estimation of the amount of siRNAs escaping from the endosome helps us to research the biological mechanism of endosomal escape more definitively. Second, the knowledge of the amount of siRNAs of escape in RNAi processes is important to design rationally the strategies that optimize the amount of siRNA to interfere with the target RNA. Finally, the amount of escape impacts the levels of the types that can escape from the endosome into the cytosol when we inject multiple types of siRNA and thereby, impact the effect of interference.

Bottleneck has been extensively researched by many articles that mostly focus on the qualitative analysis of transmission bottleneck sizes,^14^ and Abel et al.^15^ provide a biologically motivated introduction to bottlenecks. Sobel et al.^16^ use the deep-sequencing data to construct the likelihood expression of transmission bottleneck on the basis of the beta-binomial sampling method. Inspired by the above opinions with bottleneck, new ideas aiming at gauging the escaping amounts of siRNA for a single type and multiple types are suggested, respectively. After the observed data are simulated by the Gillespie algorithm, the probability distributions of escaping amounts of siRNA are estimated by means of two algorithms, consisting of the Bayesian approach and the nearest neighbor method.^17^ However, both algorithms are inefficient in the course of actual implementation, because the multiple invoking and running of the Gillespie algorithm take much time. So we provide an alternative approach to sample the escaping amounts of siRNA based on the Markov chain Monte Carlo (MCMC) method and take the means of samples as the estimation of escaping amounts to improve the speed of the computer. Finally, comparisons indicate that the estimations inferred by both Bayesian and MCMC methods approximate the true value.

## Results

### Silence Gene Controlling the Synthesis of Chitin

The oriental migratory locust is a crucial pest in agriculture.^18^ Recently, the locust plague has broken out more frequently and severely in China.^19^ As we know, the growth and development of locust strictly depend on the biosynthesis and degradation of chitin, which is absent in plants and vertebrates. So, chitin metabolism represents an attractive target for developing safe and effective insecticides.^20^

RNAi can be used to silence genes that control the synthesis of chitin, sequentially leading to the death of locusts. After siRNAs are injected into the locust, they are governed by stochastic processes, including amplification, degradation, immigration, and emigration, which are dominated by a parameter set θ={α,λ,μ,σ}. Let S(t) be the amount of the current siRNAs. Then, four stochastic processes are modeled by four biochemical reactions as follows:

(Equation 1) $$\begin{array}{cc} S\overset{\alpha }{\to }2S & \text{(a)}\\ S\overset{\lambda }{\to }\emptyset & \text{(b)}\\ \emptyset \overset{\mu }{\to }S & \text{(c)}\\ S\overset{\sigma }{\to }\emptyset & \text{(d)} \end{array}.$$

Next, the biological significance of the construction and parameters in Equation 1 are presented.

• α is the amplification rate of siRNAs that have escaped. Equation 1a means that given the current amount S(t), a unit of new siRNA is generated in the time interval (t,t+dt) with probability αS(t)dt.
• λ is the degradation rate of siRNA due to the endocytosis. Equation 1b, represents that a unit of siRNA is degraded by lysosomes with probability λS(t)dt in the time interval (t,t+dt) for given the current states S(t).
• μ is the immigration rate of a new siRNA molecule. Equation 1c reveals that a unit of siRNA immigrates in our system from the neighboring cells with probability μdt in the time interval (t,t+dt).
• σ is the emigration rate of siRNA. Equation 1d shows that siRNA will decrease one unit with the emigration of siRNA into the neighboring cells in the time interval (t,t+dt) with the probability σS(t)dt for the given current state S(t).

**Figure 2:**
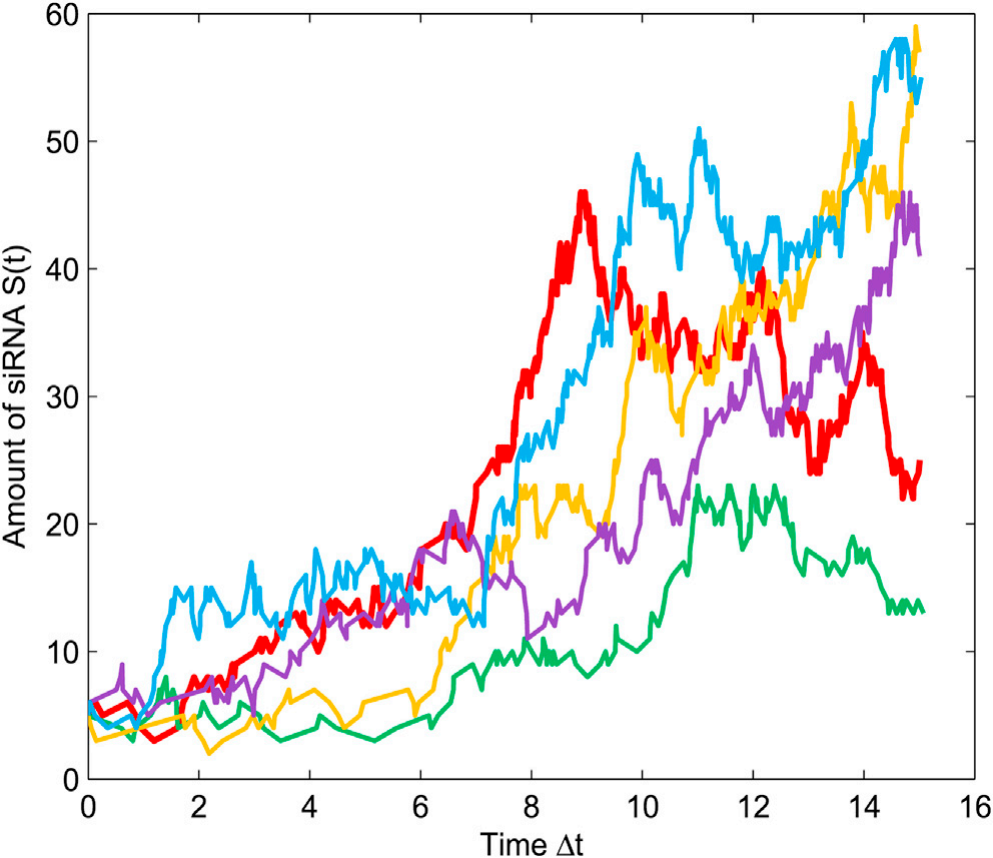
Time Evolutions of the siRNA The simulations for the dynamic of the siRNA by the Gillespie algorithm^21^ are illustrated and the lines with five different colors represent five simulations.

Take the parameter values α=0.6,λ=0.3,μ=0.6,σ=0.23, for example, when the initial value is given by S(0)=5, simulations for the dynamic of the siRNA by the Gillespie algorithm are illustrated in Figure 2. So, the value at Δt=12h could be recorded as our observation data $s_{2}$ being the amount of siRNA after amplification.

Next, the above observation data $s_{2}$ are employed to estimate the amount of escape $s_{1}$ or its posterior distribution $p\left(s_{1}|s_{2}\right)$ and meanwhile, demonstrate the efficacy of Algorithm 1 and Algorithm 2 for the single type of siRNA.

1. Given the target amount of escape $s_{1}^{*}\in \left\{1,3,5,7,70,140,700\right\}$.
2. Get the data $\left\{{\left(s_{2}\right)}_{1},{\left(s_{2}\right)}_{2},...,{\left(s_{2}\right)}_{101}\right\}\overset{i.i.d}{∼}Gillespie\left(s_{1}^{*},\Delta t,\theta \right)$.
3. Make $s_{2}^{*}$ be the median of ${\left\{{\left(s_{2}\right)}_{j}\right\}}_{j=1,...,101}$.
4. Acquire $p\left(s_{1}|s_{2}^{*}\right)$ by Algorithm 1 and the mean $s_{1}$ of samples by Algorithm 2, respectively.
5. Compare $p\left(s_{1}|s_{2}^{*}\right)$ and the mean with target $s_{1}^{*}$, respectively.

Algorithm 1 **Estimation of Probability Distributions p(s1|s2)**

**Input**: the amount of siRNAs after amplification $s_{2}$, time interval Δt, and the parameter set θ.

**Output**: the probability $p\left(s_{1}|s_{2}\right)$ when $s_{1}=1,...,s_{max}\left(s_{max}\leq s_{2}\right)$.

1. **For** $s_{1}=1$ to $s_{max}$, **do**
2.  Simulate $\left\{{\left(s_{2}\right)}_{1},{\left(s_{2}\right)}_{2},...,{\left(s_{2}\right)}_{100}\right\}$ from $s_{1}$ by the Gillespie algorithm
3.  Get $\hat{p}\left(s_{2}|s_{1}\right)$ from ${\left\{{\left(s_{2}\right)}_{j}\right\}}_{j=1,...,100}$ by the nearest neighbor method
4.  Set $prob=\hat{p}\left(s_{2}|s_{1}\right)$.
5. Set $\left[\hat{p}\left(s_{1}=1|s_{2}),...,\hat{p}(s_{1}=s_{max}|s_{2}\right)\right]=\frac{prob}{sum\left(prob\right)}$.
6. **Return** $\left[\hat{p}\left(s_{1}=1|s_{2}),...,\hat{p}(s_{1}=s_{max}|s_{2}\right)\right]$.

**Figure 3:**
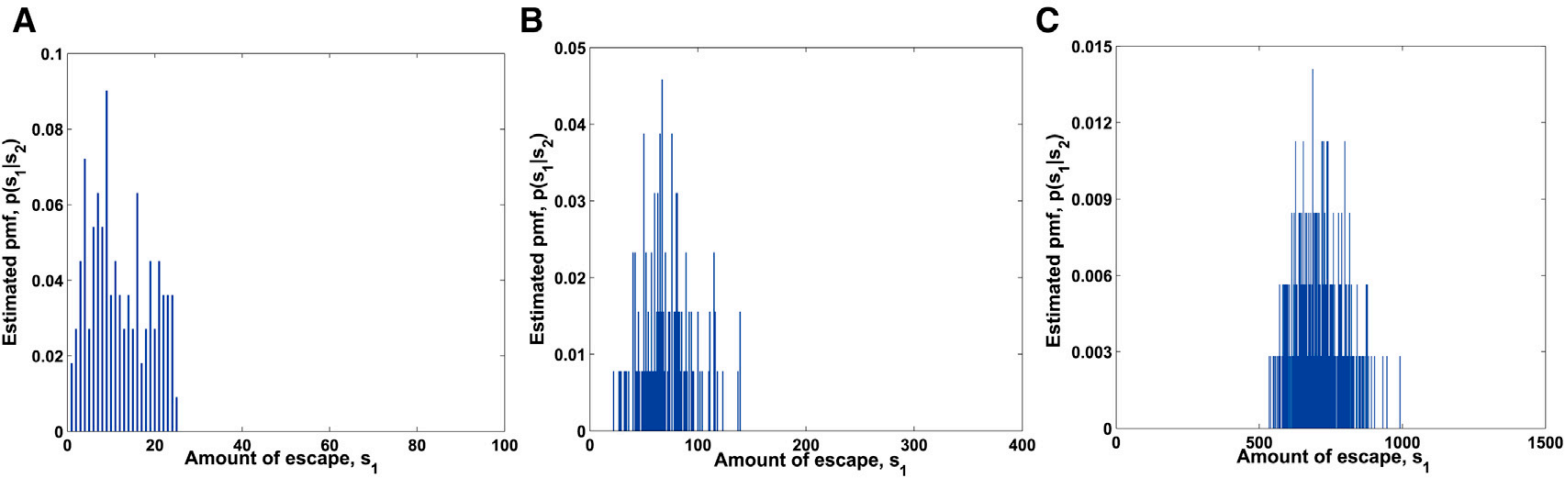
Posterior Distributions p(s_1_|s_2_) Estimated by Bayesian Inference Posterior distributions $p\left(s_{1}|s_{2}\right)$ of amount of escape estimated using Algorithm 1 for (A) $s_{1}^{*}=7$, (B) $s_{1}^{*}=70$, and (C) $s_{1}^{*}=700$.

**Figure 4:**
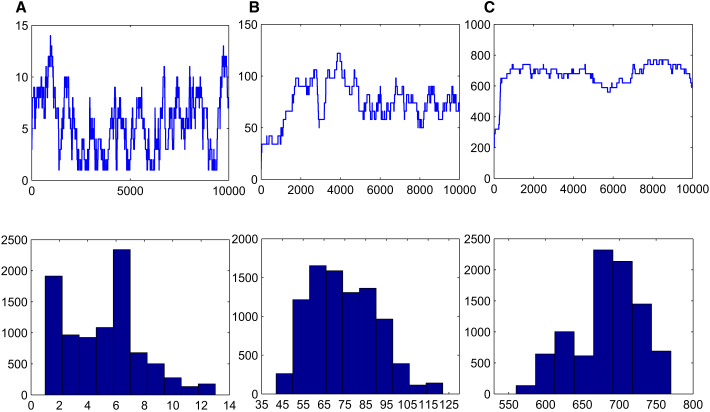
The Results of Sampling for Single Type of siRNA Obtained by MCMC Method The three panels at the top visualize the sampled data of $s_{1}$. For all other panels, the posterior distributions $p\left(s_{1}|s_{2}\right)$ obtained using Algorithm 2 are delineated. (A) refers to the target $s_{1}^{*}=7$, (B) to the $s_{1}^{*}=70$, and (C) to $s_{1}^{*}=700$.

For targets $s_{1}^{*}=7,s_{1}^{*}=70$, and $s_{1}^{*}=700$, we obtained the posterior distributions $p\left(s_{1}|s_{2}\right)$ of the escaping amount by Algorithm 1 in Figures 3A–3C. Furthermore, we take their modes 9, 67, and 687 as the estimations of the escaping amount, respectively. For the same targets, the samples of the escaping amount are displayed in Figures 4A–4C by Algorithm 2, and their means are estimated as 5, 78, and 687 after burn-in. Obviously, the two kinds of estimations fit the targets very well. This indicates that the two algorithms are efficient.

Algorithm 2 **Generating the Samples of s1**

**Input**: the amount of siRNAs after amplification $s_{2}$, time interval Δt, the parameter set θ, initial value $s_{1}^{\left(0\right)}$, number of iterations N, and cycle index k=0.

**Output**: the sample $s_{1}^{\left(0\right)},s_{1}^{\left(1\right)},...,s_{1}^{\left(N\right)}$.

1. Simulate $s_{2}^{\left(k\right)}$ from $s_{1}^{\left(k\right)}$ by the Gillespie algorithm, and calculate $d=\left|s_{2}^{\left(k\right)}-s_{2}\right|$
2. **For** k=0 to N, **do**
3.  Generate a proposed value $s_{1}^{'}$ from proposal distribution $q\left(s_{1}^{\text{'}}|s_{1}^{\left(k\right)}\right)$
4.  Simulate $s_{2}^{'}$ from $s_{1}^{'}$ by the Gillespie algorithm, and calculate $d'=\left|s_{2}^{'}-s_{2}\right|$
5.  Sample u from uniform distribution U(0,1)
6.  Calculate the acceptance probability α by (Equation 7)
7.  **If** $u\leq \alpha \left(s_{1}^{'},s_{1}^{\left(k\right)}\right)$, **then**
8.  Accept $s_{1}^{'}$, and set $s_{1}^{\left(k+1\right)}=s_{1}^{'},d=d^{\prime }$
9.  **else**
10.  Reject $s_{1}^{'}$, and set $s_{1}^{\left(k+1\right)}=s_{1}^{\left(k\right)},d=d$
11. **Return** $s_{1}^{\left(0\right)},s_{1}^{\left(1\right)},...,s_{1}^{\left(N\right)}$

### Interfere Multiple Target Oncogene

Related studies have found that the cancerization of normal cells is the consequence of interaction of multiple genes. However, conventional therapies, which are only targeted toward a single gene mostly, cannot completely inhibit the growth of tumors. It is obvious that RNAi technology can be utilized to silence gene. Yin et al.^22^ suggested that injecting multiple types of siRNA can specifically interfere with multiple target oncogenes simultaneously and thereby inhibit the growth and proliferation of cancer cells synergistically.

Consequently, for multiple types, a hypothesis is given that we inject seven types of siRNA $v_{0}^{\left[1\right]},v_{0}^{\left[2\right]},...,v_{0}^{\left[7\right]}$ for gene therapy. Then, the observation data $v_{2}$ could be simulated by the Gillespie algorithm, as previously mentioned. Algorithm 3 and Algorithm 4 are applied to estimate the amount of escaping siRNAs and verify the efficacy of these two methods by the following steps.

1. Given the initial injected amount, $v_{0}=\langle 600^{\left[1\right]},600^{\left[2\right]},...,600^{\left[7\right]}\rangle$.
2. Given the target amount of escape, $s_{1}^{*}\in \left\{1,3,5,7,70,140\right\}$.
3. Generate a mode $v_{1}^{*}$ using the multivariate hypergeometric distribution related to random samples of size $s_{1}^{*}$ from $v_{0}$.
4. Get the data $\left\{{\left(v_{2}\right)}_{1},{\left(v_{2}\right)}_{2},...,{\left(v_{2}\right)}_{101}\right\}\overset{i.i.d}{∼}Gillespie\left(v_{1}^{*},\Delta t,\theta \right)$.
5. Then, make $v_{2}^{*}$ be the median of ${\left\{{\left(v_{2}\right)}_{j}\right\}}_{j=1,...,101}$.
6. Acquire $p\left(s_{1}|v_{0},v_{2}\right)$ by Algorithm 3 and the mean $s_{1}$ of samples by Algorithm 4, respectively.
7. Compare $p\left(s_{1}|v_{0},v_{2}\right)$ and the mean with target $s_{1}^{*}$, respectively.

Algorithm 3 **Estimation of Probability Distributions p(s1|v0,v2)**

**Input**: the initial injected amount of siRNAs of various types $v_{0}$, the amount of siRNAs after amplification $v_{2}$, time interval Δt, and the parameter set θ.

**Output**: the probability $p\left(s_{1}|v_{0},v_{2}\right)$ when $s_{1}=1,...,s_{max}$.

1. **For** $s_{1}=1$ to $s_{max}$, **do**
2.  **For** k=1 to 1,000, **do**
3.  Sample $v_{1}$ from the multivariate hypergeometric distribution with $s_{1}$, $v_{0}$
4.  Calculate $p\left(v_{1}|v_{0}\right)$ by (Equation 11)
5.  Set $a=p\left(v_{1}|v_{0}\right)$
6.  Set b=1
7.  **For** $v_{1}^{\left[i\right]}\in \;v_{1}$, **do**
8.  Simulate $\left\{{\left(v_{2}^{\left[i\right]}\right)}_{1},{\left(v_{2}^{\left[i\right]}\right)}_{2},...,{\left(v_{2}^{\left[i\right]}\right)}_{100}\right\}$ from $v_{1}^{\left[i\right]}$ by the Gillespie algorithm
9.  Get $\hat{p}\left(v_{2}^{\left[i\right]}|v_{1}^{\left[i\right]}\right)$ from ${\left\{{\left(v_{2}^{\left[i\right]}\right)}_{j}\right\}}_{j=1,...,100}$ by the nearest neighbor method
10.  Set $p=\hat{p}\left(v_{2}^{\left[i\right]}|v_{1}^{\left[i\right]}\right)$
11.  Set b=b×p ▹b is $\hat{p}\left(v_{2}|v_{1}\right)$ at last
12.  Set prob=a×b
13.  Set numert=sum(prob)
14. Set $\left[\hat{p}\left(s_{1}=1|v_{0},v_{2}),\ldots ,\hat{p}(s_{1}=s_{max}|v_{0},v_{2}\right)\right]=\frac{numert}{sum\left(numert\right)}$
15. Get the modes of $\left[\hat{p}\left(s_{1}=1|v_{0},v_{2}),...,\hat{p}(s_{1}=s_{max}|v_{0},v_{2}\right)\right]$ as an estimation of $s_{1}$
16. **Return** $\left[\hat{p}\left(s_{1}=1|v_{0},v_{2}),...,\hat{p}(s_{1}=s_{max}|v_{0},v_{2}\right)\right]$

**Figure 5:**
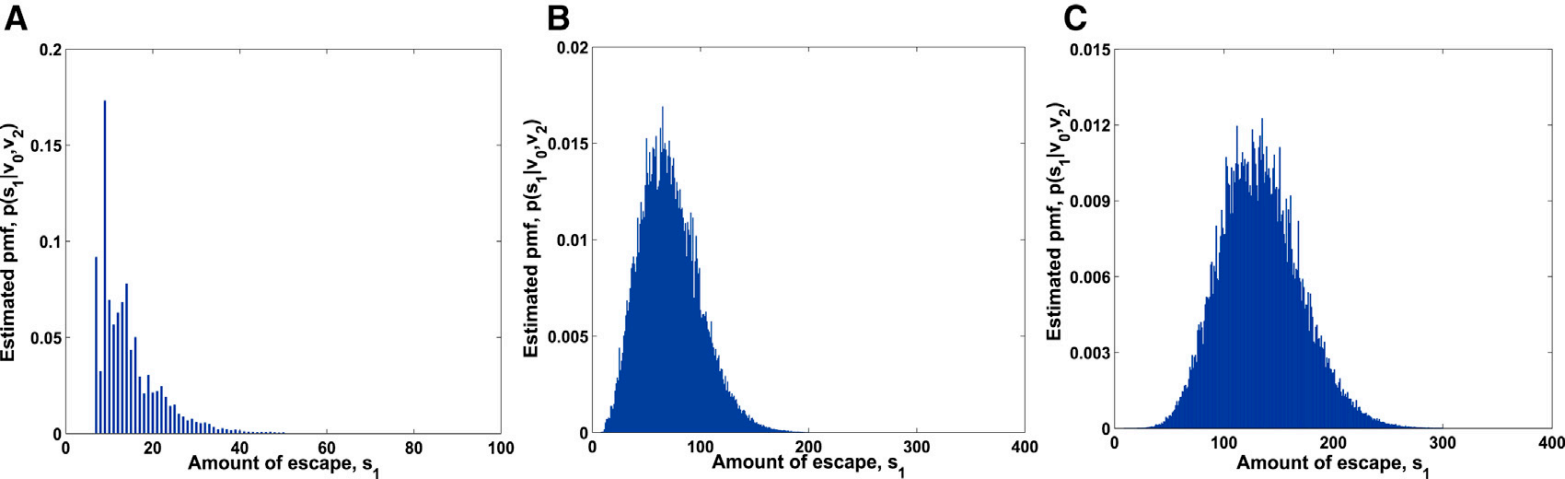
Posterior Distributions p(s_1_|**v_0_**,**v_2_**) Estimated by Bayesian Inference Posterior distributions of amount of escape estimated using Algorithm 3 for (A) $s_{1}^{*}=7$, (B) $s_{1}^{*}=70$, and (C) $s_{1}^{*}=140$.

**Figure 6:**
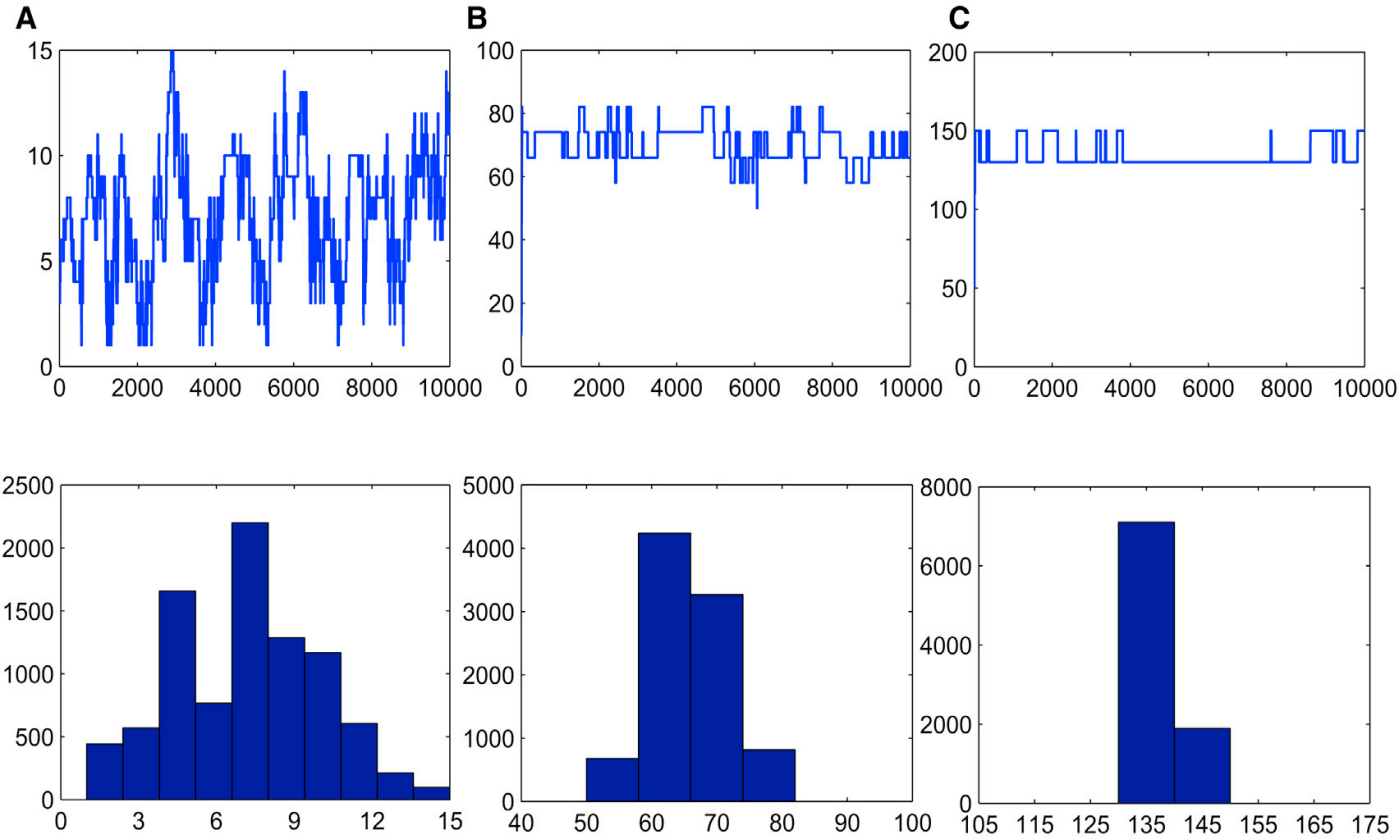
The Results of Sampling for Multiple Types of siRNAs Obtained by MCMC Method The three panels at the top visualize the sampled data of $s_{1}$. For all other panels, the posterior distributions $p\left(s_{1}|v_{0},v_{2}\right)$ obtained using Algorithm 4 are delineated. (A) refers to the target $s_{1}^{*}=7$, (B) to the $s_{1}^{*}=70$, and (C) to $s_{1}^{*}=140$.

Estimated posterior distributions $p\left(s_{1}|v_{0},v_{2}\right)$ by Algorithm 3 are shown in Figures 5A–5C for targets $s_{1}^{*}=7,s_{1}^{*}=70$, and $s_{1}^{*}=140$. The modes, as the estimations of the escaping amount, are 9, 65, and 135, respectively. For the same targets, we perform 10,000 samples by Algorithm 4 and report in Figures 6A–6C. After burn-in, we get the estimations 9, 69, and 133 by calculating their means. It can be seen that our predicted results approximate accurately to real ones.

Algorithm 4 **Generating the Samples of s1**

**Input**: the initial injected amount of siRNAs $v_{0}$, the amount of siRNAs after amplification $v_{2}$, time interval Δt, parameter set θ, initial value $s_{1}^{\left(0\right)}$, number of iterations N, and cycle index k=0.

**Output**: the samples $s_{1}^{\left(0\right)},s_{1}^{\left(1\right)},...,s_{1}^{\left(N\right)}$.

1. Sample $v_{1}^{\left(k\right)}$ from the multivariate hypergeometric distribution with $v_{0}$, $s_{1}^{\left(k\right)}$
2. Simulate $v_{2}^{\left(k\right)}$ from $v_{1}^{\left(k\right)}$ using the Gillespie algorithm, and calculate $d=\left\|v_{2}^{\left(k\right)}-v_{2}\right\|$
3. **For** k=0 to N, **do**
4.  Generate a proposed value $s_{1}^{'}$ from proposal distribution $q\left(s_{1}^{\text{'}}|s_{1}^{\left(k\right)}\right)$
5.  Sample $v_{1}^{'}$ from the multivariate hypergeometric distribution with $v_{0}$, $s_{1}^{'}$
6.  Simulate $v_{2}^{'}$ from $v_{1}^{'}$ by the Gillespie algorithm, and calculate $d^{\prime }=\left\|v_{2}^{'}-v_{2}\right\|$
7.  Sample u from uniform distribution U(0,1)
8.  Calculate the acceptance probability α by (Equation 17)
9.  **If** $u\leq \alpha \left(s_{1}^{'},s_{1}^{\left(k\right)}\right)$, **then**
10.  Accept $s_{1}^{'}$, and set $s_{1}^{\left(k+1\right)}=s_{1}^{'},d=d^{\prime }$
11.  **else**
12.  Reject $s_{1}^{'}$, and set $s_{1}^{\left(k+1\right)}=s_{1}^{\left(k\right)},d=d$
13. **Return** $s_{1}^{\left(0\right)},s_{1}^{\left(1\right)},...,s_{1}^{\left(N\right)}$

## Discussion

The amount of siRNAs escaping from the endosome is one of the important essentials dominating the efficiency of RNAi, but it is intractable to be observed and calculated in experiments. In this paper, two methods are proposed to estimate the amount of escape in terms of the knowledge of the dynamics during amplification from the amount after the reaction and the amount of injection. One is to estimate the posterior distribution of escaping the amount according to the Bayesian approach; the other one is to get the samples of the escaping amount by the MCMC method and to use the mean of samples as an estimate. For the traditional Bayesian approach, we present the specific algorithms combined with the nearest neighbor method, which is used for the estimation of $p\left(s_{2}|s_{1}\right)$. For the MCMC method, the acceptance probability of the Metropolis-Hastings (MH) algorithm is controlled by the distance function between the simulation with the observed data. Furthermore, with the contraposition of the single type of siRNAs and multiple types of siRNAs, the algorithms of the estimate of the escaping amount are given, respectively. To inspect the validity of our algorithms, two examples on the silencing gene for the synthesis of chitin and blocking multiple target oncogenes are derived. Our pursuit offers statistical ways to infer the exact amount of siRNAs participating in the actual RNAi reaction. Meanwhile, it perhaps provides a theoretical basis to decrease the cost of the biotic experiment for the future.

Even so, there are still some problems worth exploring further. First, the MCMC method failed to estimate the posterior distribution that could express the uncertainty through the variance of the distributions, although it improves the efficiency. It indicates that a more comprehensive method that takes into account the accuracy of estimation, efficiency, and expression of uncertainty together is required. Besides, the estimation of the bottleneck size is only built on the assumption that the dynamics during amplification are known. When the partial data are missing, how to estimate the amount of escape and the parameters together is the problem for further consideration. In future research, we will try to find the solutions to these problems.

## Materials and Methods

### Single Type of siRNA

**Figure 7:**
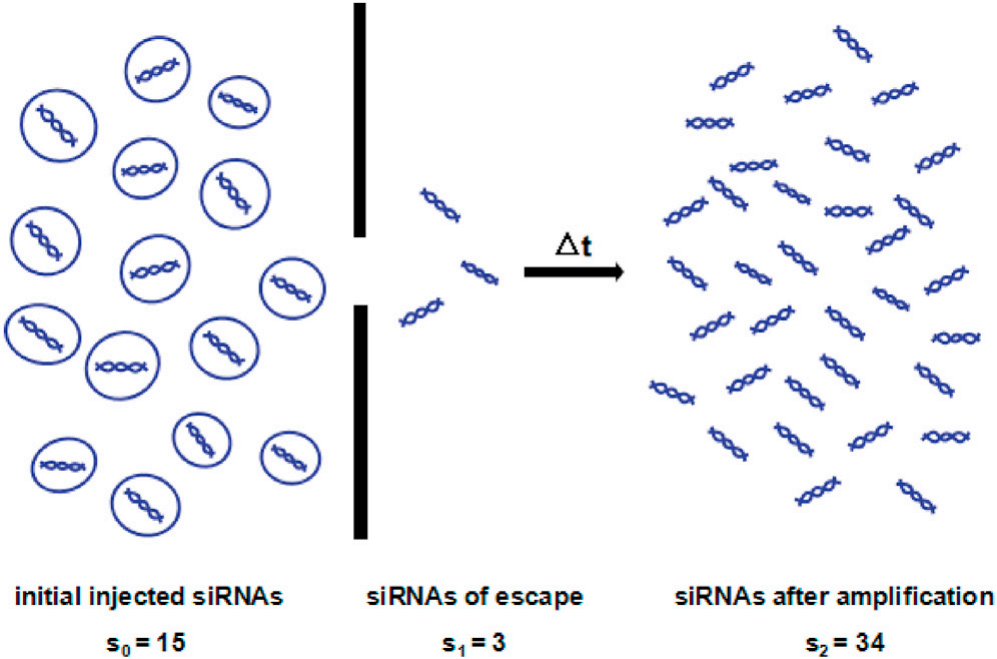
Diagrammatic Representation of the Process that siRNAs Escape and the Amount at Each Stage Firstly, the siRNAs with an initial injected amount s_0_ escape from endosome. And then, the escaping siRNAs s_1_ are amplified to s_2_ after Δt time.

In general, we only introduce a single type of siRNA aimed at a specific RNA into the organisms. The processes for which siRNAs escape from the endosome and amplify intracellularly have been described in the first part, and now, we picture them in Figure 7. Define the initial injected amount of siRNAs as $s_{0}$, the amount of siRNAs that escape from endosome as $s_{1}$, and the amount of siRNAs after amplification as $s_{2}$ (Figure 7). Obviously, $s_{1}\leq s_{2}$. Then, on the premise of the amount of siRNAs after amplification, Bayesian inference or MCMC can be applied to estimate the posterior distribution of the escaping amount of siRNAs, as well as their value.

#### Bayesian Inference

According to the Bayesian framework, the amount of siRNAs escaping from the endosome can be estimated by the posterior probability distributions. Given the observations of the amount after amplification, the distribution is given by

$$p\left(\text{amount}\;\text{of}\;\text{escape}\;\left(s_{1}\right)|\text{amount}\;\text{after}\;\text{amplification}\;\left(s_{2}\right)\right).$$

The merit of the use of the Bayesian approach is that we not only could get the estimates of the most probable amount of escape (in terms of the modes of the distribution), but also, we could be aware of the uncertainty via the variance of the distributions. Then, the posterior probability $p\left(s_{1}|s_{2}\right)$ is given by

(Equation 2) $$p\left(s_{1}|s_{2}\right)=\frac{p\left(s_{1}\right)p\left(s_{2}|s_{1}\right)}{\sum_{s_{1}}p\left(s_{1}\right)p\left(s_{2}|s_{1}\right)}\propto p\left(s_{1}\right)p\left(s_{2}|s_{1}\right).$$

With the further assumption of the prior $p\left(s_{1}\right)$ to be equally likely, one gets

(Equation 3) $$p\left(s_{1}|s_{2}\right)=\frac{p\left(s_{2}|s_{1}\right)}{\sum_{s_{1}}p\left(s_{2}|s_{1}\right)}.$$

Then, the posterior distribution $p\left(s_{1}|s_{2}\right)$ can be obtained through estimating all of the probability $p\left(s_{1}|s_{2}\right)$ for $s_{1}=1,...,s_{max}$, where $s_{max}$ is the maximum of escaping amount $s_{1}$. The detailed process is shown as follows.

First, starting from $s_{1}$, we perform n simulations using the Gillespie stochastic algorithm,^21^ according to a parameter set θ for the dynamics, and obtain the finite simulating samples of $s_{2}$ after time interval Δt:

$$\left\{{\left(s_{2}\right)}_{1},{\left(s_{2}\right)}_{2},...,{\left(s_{2}\right)}_{n}\right\},$$

from which $p\left(s_{2}|s_{1},\theta \right)$ is estimated using the nearest neighbor method,^17^ which is a classical nonparametric estimation method.

Second, with the substitution of all probabilities $p\left(s_{2}|\cdot ,\cdot \right)$ into Equation 3, one gets the estimation of the probability distribution $p\left(s_{1}|s_{2}\right)$.

In detail, the algorithm for estimating distribution $p\left(s_{1}|s_{2}\right)$ is given as follows.

Algorithm 1 implies that the Gillespie algorithm runs n times when the loop executes one time. It reveals that Algorithm 1 is time consuming if simulating time n is large. So, in order to improve the running efficiency of program, we adopt the MCMC method to estimate the escaping amount of siRNAs.

#### MCMC Method

The MCMC method includes Gibbs and MH, which are techniques simulating the random variables by using the Markov chain.^23^ In this paper, we choose MH to sample single variable $s_{1}$, rather than Gibbs from the target distribution, being the conditional distribution of interest. Here, the target distribution, that is, posterior distribution $p\left(s_{1}|s_{2}\right)$ in Equation 2, is proportional to the product of prior $p\left(s_{1}\right)$ and likelihood $p\left(s_{2}|s_{1}\right)$.

From the ideas of MCMC, we need to compute the acceptance probability $\alpha \left(s_{1}^{\text{'}},s_{1}^{\left(k\right)}\right)$,

where $s_{1}^{\left(k\right)}$ is k th sample, and $s_{1}^{'}$ is a proposed value. From the symmetry of proposal distribution, namely $q\left(s_{1}^{'}|s_{1}^{\left(k\right)})=q(s_{1}^{\left(k\right)}|s_{1}^{'}\right)$,^24^ and equally likely possibility of prior

$p\left(s_{1}\right)$, the acceptance probability can be simplified to

(Equation 4) $$\alpha \left(s_{1}^{\text{'}},s_{1}^{\left(k\right)}\right)=\text{min}\left\{1,\frac{p(s_{2}|s_{1}^{'})}{p(s_{2}|s_{1}^{\left(k\right)})}\right\}.$$

Again, because $\text{p}\left(s_{2}|{\text{s}}_{1}^{\text{'}}\right)$ and $\text{p}\left(s_{2}|{\text{s}}_{1}^{\left(\text{k}\right)}\right)$ in Equation 4 are unknown, next, we pursue a novel approach to compute them. For $\text{p}\left(s_{2}|{\text{s}}_{1}^{\left(k\right)}\right)$, first of all, we simulate one value $s_{2}^{\left(k\right)}$ from $s_{1}^{\left(k\right)}$ after a certain time Δt by the Gillespie algorithm. Second, we compute the distance between the given value $s_{2}$ and the simulation $s_{2}^{\left(k\right)}$ denoted by $d=\left|s_{2}^{\left(k\right)}-s_{2}\right|$. Finally, the likelihood^25^ is calculated by

(Equation 5) $$p\left(s_{2}|s_{1}^{\left(k\right)}\right)=e^{-d}.$$

Similarly, another likelihood in Equation 4 is calculated by

(Equation 6) $$p\left(s_{2}|s_{1}^{'}\right)=e^{-d'},$$

where $d'=\left|s_{2}^{'}-s_{2}\right|$, while $s_{2}^{'}$ is simulating from $s_{1}^{'}$ by the same way as $s_{2}^{\left(k\right)}$.

From all of the above, the acceptance probability in Equation 4 is renovated by

(Equation 7) $$\alpha \left(s_{1}^{'},s_{1}^{\left(k\right)}\right)=min\left\{1,\frac{e^{-d^{\prime }}}{e^{-d}}\right\}.$$

Now, the procedure of sampling $s_{1}$ by MCMC methods is listed as follows.

### Multiple Types of siRNA

**Figure 8:**
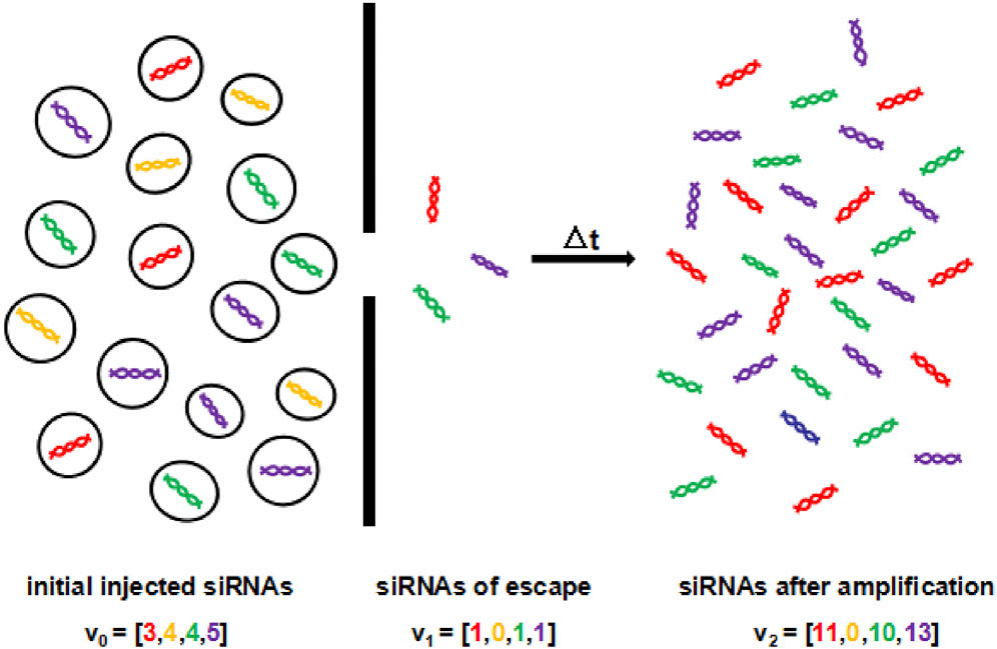
Diagrammatic Representation of the Process in which siRNAs Escape and Their Propensity to Stochastic Variability in Terms of Both the Amount and the Composition of Their Population Different colors express different types of siRNAs. Initial injected multiple types of siRNA consist of **v_0_**. After endocytosis, their amount decline to **v_1_**. Subsequently, the escaping siRNAs are amplified to **v_2_** after Δt time.

With the consideration of injecting multiple types of siRNAs to affect different target RNAs, the stochastic process of siRNAs is shown in Figure 8. Assume that we inject m types of siRNA for which the initial injected amount consists of $v_{0}^{\left[1\right]},v_{0}^{\left[2\right]},...,v_{0}^{\left[m\right]}$, where $v_{0}^{\left[i\right]}\geq 0$ is the amount of i th siRNA. The amount of siRNA is declined to the relatively lower values of $v_{1}^{\left[1\right]},v_{1}^{\left[2\right]},...,v_{1}^{\left[m\right]}$ because of endocytosis. After amplification, the composition of siRNA develops into $v_{2}^{\left[1\right]},v_{2}^{\left[2\right]},...,v_{2}^{\left[m\right]}$ (Figure 8). With initial injected amount $v_{0}=\left(v_{0}^{\left[1\right]},v_{0}^{\left[2\right]},...,v_{0}^{\left[m\right]}\right)$ and the amount $v_{2}=\left(v_{2}^{\left[1\right]},v_{2}^{\left[2\right]},...,v_{2}^{\left[m\right]}\right)$ after amplification known, the Bayesian inference and MCMC method can be applied to estimate the posterior distribution $p\left(s_{1}|v_{0},v_{2}\right)$ and sample $s_{1}$ from this posterior distribution, respectively.

#### Bayesian Inference

For the posterior distribution $p\left(s_{1}|v_{0},v_{2}\right)$, we have

(Equation 8) $$\begin{array}{c} p\left(s_{1}|v_{0},v_{2})=p(\underset{\underset{s.t.sum\;\left(v_{1}\right)=s_{1}}{v_{1}}}{\vee }v_{1}|v_{0},v_{2}\right)\\ =\sum_{\underset{s.t.sum\;\left(v_{1}\right)=s_{1}}{v_{1}}}p\left(v_{1}|v_{0},v_{2}\right) \end{array},$$

where $sum\left(v_{1}\right)=\sum_{i}v_{1}^{\left[i\right]}$. Again, from Bayes’ theorem, one gets

(Equation 9) $$\begin{array}{c} p\left(v_{1}|v_{0},v_{2}\right)=\frac{p\left(v_{1}|v_{0}\right)p\left(v_{2}|v_{1},v_{0}\right)}{\sum_{v_{1}}p\left(v_{1}|v_{0}\right)p\left(v_{2}|v_{1},v_{0}\right)}\\ =\frac{p\left(v_{1}|v_{0})p(v_{2}|v_{1}\right)}{\sum_{v_{1}}p\left(v_{1}|v_{0})p(v_{2}|v_{1}\right)} \end{array}.$$

Therefore, the incorporation of Equations 8 and 9 yields

(Equation 10) $$p\left(s_{1}|v_{0},v_{2}\right)=\frac{\sum_{\begin{array}{c} v_{1}\\ s.t.sum(v_{1})=s_{1} \end{array}}p(v_{1}|v_{0})p\left(v_{2}|v_{1}\right)}{\sum_{v_{1}}p\left(v_{1}|v_{0}\right)p\left(v_{2}|v_{1}\right)}=\frac{\sum_{\begin{array}{c} v_{1}\\ s.t.sum\left(v_{1}\right)=s_{1} \end{array}}p\left(v_{1|}v_{0}\right)p\left(v_{2}|v_{1}\right)}{\sum_{s1}\;\;\;\;\;\sum_{\begin{array}{c} v_{1}\\ s.t.sum\left(v_{1}\right)=s_{1} \end{array}}p\left(v_{1|}v_{0}\right)p\left(v_{2|}v_{1}\right)}$$

Assume that all types of siRNAs are phenotypically identical and have the same probability of escaping from the endosome. Then, the distribution $v_{1}^{\left[1\right]},v_{1}^{\left[2\right]},...,v_{1}^{\left[m\right]}$ of $s_{1}$ could be considered as sampling randomly without replacement from the initial injected amount with distribution $v_{0}$. So, we can select the amount of escape from a multivariate hypergeometric distribution with $v_{0}$ and $sum\text{(}v_{1})=s_{1}$. The probability of drawing $v_{1}$ from $v_{0}$ is given by

(Equation 11) $$p\left(v_{1}|v_{0}\text{, }sum\left(v_{1}\right)=s_{1}\right)=\frac{\left(\begin{array}{c} v_{0}^{\left[1\right]}\\ v_{1}^{\left[1\right]} \end{array}\right)\left(\begin{array}{c} v_{0}^{\left[2\right]}\\ v_{1}^{\left[2\right]} \end{array}\right)...\left(\begin{array}{c} v_{0}^{\left[m\right]}\\ v_{1}^{\left[m\right]} \end{array}\right)}{\left(\begin{array}{c} v_{0}^{\left[1\right]}+v_{0}^{\left[2\right]}+...+v_{0}^{\left[m\right]}\\ v_{1}^{\left[1\right]}+v_{1}^{\left[2\right]}+...+v_{1}^{\left[m\right]} \end{array}\right)}.$$

In reality, components of $v_{2}$ are simulated by the Gillespie algorithm in view of parameter vector θ. So, for convenience, $p\left(v_{2}|v_{1}\right)$ is denoted by $p\left(v_{2}|v_{1},\theta \right)$, which is factorized in accordance with the independence between each type of siRNA as follows:

(Equation 12) $$p\left(v_{2}|v_{1},\theta \right)=\prod_{i}p\left(v_{2}^{\left[i\right]}|v_{1},\theta \right)=\prod_{i}p\left(v_{2}^{\left[i\right]}|v_{1}^{\left[i\right]},\theta \right).$$

Then, $p\left(v_{2}^{\left[i\right]}|v_{1}^{\left[i\right]},\theta \right)$ could be estimated the same way that we estimate $p\left(s_{2}|s_{1},\theta \right)$, used in Algorithm 1.

The acquisition of $p\left(v_{1}|v_{0}\right)$ and $p\left(v_{2}|v_{1}\right)$ that are desired for Equation 10 has been solved in the previous segment, but we should count all of the summands when $v_{2}$ gets every possible value, such that $\sum_{i}v_{1}^{\left[i\right]}=s_{1}$. One key problem is that all possible values of $v_{1}$ grow superexponentially with $s_{1}$ when we give a value of $s_{1}$.^26^ Now, we face a combinatorial and computational challenge, and so a replaceable approach is required.

To avoid the combinatorial problem, the more probable configuration of $v_{1}$, such as the modes of $v_{1}$, could replace the summands that consider all possibilities of $v_{1}$ in Equation 10. Requena et al.^27^ have elaborated an algorithm to solve this question, but now, we provide a simpler sampling method that is to sample points $v_{1}$ randomly from multivariate hypergeometric distribution $p\left(v_{1}|v_{0}\right)$ for enough times so that most of these points would be adjacent to the modes. The concrete execution of the sampling procedure is shown in Algorithm 3.

Likewise, as discussed in the context above, there are problems of efficiency with this approach. Therefore, it is tempting to attempt to use the MCMC method.

#### MCMC Method

Multiple types are also appropriate for the MCMC method. Similar to the single type, our target distribution is posterior distribution $p\left(s_{1}|v_{0},v_{2}\right)$ now. From Equation 9, we get

(Equation 13) $$p\left(s_{1}|v_{0},v_{2}\right)\propto p\left(s_{1}|v_{0}\right)p\left(v_{2}|s_{1}\right).$$

In view of the equal possibility of the prior $p\left(s_{1}\right)$ and the previous Equation 13, the acceptance probability about the MH method is given by

(Equation 14) $$\alpha \left(s_{1}^{'},s_{1}^{\left(k\right)}\right)=min\left\{1,\frac{p(v_{2}|s_{1}^{'})}{p(v_{2}|s_{1}^{\left(k\right)})}\right\}.$$

In order to go to the acceptance probability, first, we should draw $v_{1}^{'}$ from the multivariate hypergeometric distribution with $s_{1}$ and given $v_{0}$. Afterward, simulate one vector of $v_{2}^{'}$ from $v_{1}^{'}$ after Δt by the Gillespie algorithm, and then, the distance between the given value $v_{2}$ and the simulation $v_{2}^{'}$ is recorded as $d^{\prime }=\left\|v_{2}^{'}-v_{2}\right\|$. Finally, we give the numerator in Equation 14 as

(Equation 15) $$p\left(v_{2}|s_{1}^{'}\right)=e^{-d^{\prime }}.$$

Let $v_{2}^{\left(k\right)}$ be simulating from $s_{1}^{\left(k\right)}$, and $d=\left\|v_{2}^{\left(k\right)}-v_{2}\right\|$, the denominator in Equation 14, is computed by

(Equation 16) $$p\left(v_{2}|s_{1}^{\left(k\right)}\right)=e^{-d}.$$

Then, we accept $s_{1}^{'}$ with probability

(Equation 17) $$\alpha \left(s_{1}^{'},s_{1}^{\left(k\right)}\right)=min\left\{1,\frac{e^{-d^{\prime }}}{e^{-d}}\right\}.$$

The exact process of the MCMC method is described in Algorithm 4.

## Author Contributions

Y.P. conceived the project and designed the frame of this paper; T.L. and C.L. finished mathematical analyses, performed simulations and wrote the first draft; M.Y. polished, revised the last draft. All authors contributed to the manuscript and approved the final manuscript.

## Conflicts of Interest

The authors declare no competing interests.
